# Construction of high-density genetic linkage maps for orange-spotted grouper *Epinephelus coioides* using multiplexed shotgun genotyping

**DOI:** 10.1186/1471-2156-14-113

**Published:** 2013-12-01

**Authors:** Xinxin You, Liping Shu, Shuisheng Li, Jieming Chen, Jian Luo, Jun Lu, Qian Mu, Jie Bai, Qiuju Xia, Qingchun Chen, Yanjie Cai, Haifa Zhang, Guohua Chen, Haoran Lin, Yong Zhang, Qiong Shi

**Affiliations:** 1 School of Life Sciences, Sun Yat-Sen University, Guangzhou 510275, China; 2 Marine and Fisheries Institute, BGI-Shenzhen, Shenzhen 518083, China; 3 State Key Laboratory of Agricultural Genomics, BGI-Shenzhen, Shenzhen 518083, China; 4 College of Ocean, Hainan University, Haikou 570228, China; 5 Guangdong Daya Bay Fishery Development Center, Huizhou 510681, China; 6 Shenzhen BGI Fisheries Sci & Tech Co. Ltd., BGI-Shenzhen, Shenzhen 518083, China

**Keywords:** *Epinephelus coioides*, Genetic linkage map, Next generation sequencing (NGS), Multiplexed shotgun genotyping (MSG)

## Abstract

**Background:**

Orange-spotted grouper, *Epinephelus coioides*, is one of the most valuable fish species in China. Commercial production of orange-spotted grouper could be increased by developing higher growth rates and improving commercially important traits. Information on genetic markers associated with quantitative trait loci (QTL) can be used in breeding programs to identify and select individuals carrying desired traits. A high-density genetic linkage map is the basis for QTL study, and multiplexed shotgun genotyping (MSG) facilitates the development of single nucleotide polymorphisms (SNPs) and genotyping. In this study, the first high-density genetic linkage maps for groupers were generated on the basis of the MSG method.

**Results:**

The sex-averaged map contained a total of 4,608 SNPs, which spanned 1581.7 cM, with a mean distance between SNPs of 0.34 cM. The 4,608 SNPs were located in 2,849 unique locations on the linkage map, with an average inter-location space at 0.56 cM. There were 2,516 SNPs on the female map, and the number of unique locus was 1,902. However, the male map contained more numbers of SNP (2,939) and unique locations (2,005). The total length of the female and male maps was 1,370.9 and 1,335.5 cM, respectively.

**Conclusions:**

The high-resolution genetic linkage maps will be very useful for QTL analyses and marker-assisted selection (MAS) for economically important traits in molecular breeding of the orange-spotted grouper.

## Background

Grouper is the common name for a group of fishes belonging to a number of genera in the subfamily Epinephelinae of the family Serranidae, and they mainly inhabit in the Indian Ocean, the Red Sea, the Mediterranean Sea, the Northern Pacific Ocean, the Western Pacific Ocean and the Southeast Asia [1]. The orange-spotted grouper, *Epinephelus coioides*, is a valuable and popular seafood fish, and is one of the major mariculture species in China. According to FAO fishery statistics, the global aquaculture production of the orange-spotted grouper was 152 t and the value had reached to 1,246,000 US dollars in 2011 [2]. Currently in China, the orange-spotted grouper has become a major food in live fish markets and is an important cultured fish for commercial sale in Guangdong, Hainan and Fujian provinces of China. The vast market demands for orange-spotted grouper have driven efforts to breed families and populations with higher growth rates and lower food coefficients. The application of marker-assisted selection (MAS) to the orange-spotted grouper will become a promising strategy for improving growth traits [3].

A high-density genetic linkage map is the basis for quantitative trait loci (QTL) mapping, MAS, and functional gene positional cloning, and will be useful for functional genomics and genetic breeding studies. Genetic maps can also provide insights into genome organization and evolution through comparative studies. At present, high-density genetic linkage maps are already available for some important economic fishes, including Atlantic salmon [4], catfish [5], rainbow trout [6], tilapia [7], and Japanese flounder [8]. Recently, a genetic linkage map of kelp grouper (*Epinephelus bruneus*) has been established based on microsatellite markers, which is the first linkage map in the subfamily Epinephelinae [9]. However, construction of a high-density genetic linkage map for groupers has not been reported.

The density of genetic linkage map is mainly determined by the chosen genetic markers. Single nucleotide polymorphisms (SNPs) describes polymorphisms caused by point mutations at a given nucleotide position within a locus. SNPs are abundant and widely distributed throughout the genomes. As a result, SNPs have emerged as the most attractive genetic markers for construction of high-density genetic linkage map [4]. With the advent of next-generation sequencing (NGS), there are several such approaches that are capable of discovering, sequencing and genotyping thousands of SNPs across almost any genome of interest in a single step, even in species in which little or even no genetic information is available [10]. A particularly efficient protocol, termed as “multiplexed shotgun genotyping” (MSG), base on the NGS, has been used for high-throughput discovering of SNPs. In fact, MSG is similar in spirit to restriction site-associated DNA (RAD) sequencing [11] and whole-genome resequencing (WGD) [12], but combines the advantages of both techniques. MSG involves a highly simplified protocol for library preparation that requires only ~2 d of lab work to process 96 individuals or more. The technique is inexpensive because it requires only standard molecular laboratory equipment, uses only one set of bar-coded adapters, and does not require shearing and repairing of genomic DNAs. Because the approach does not depend on manual shearing, small amounts of DNA isolated from single individuals can be processed [13].

Here, we generated an orange-spotted grouper F1 full-sib family, which was genotyped by multiplexed shotgun sequencing, and at the first time reported the construction of high-density genetic linkage maps for groupers.

## Methods

### Family material and DNA isolation

An orange-spotted grouper F1 full-sib family was generated on the experimental base of Hainan Green Aquatic Breeding Co. Ltd., China, in May 2011. Fin clips of the parents were collected and kept in absolute ethanol, whereas the whole body of 142 offspring at the age of 35 days post hatch were soaked in absolute ethanol, and kept in a -20°C freezer. Genomic DNA was isolated using the standard phenol-chloroform protocol [14]. DNA quality was evaluated by Qubit Fluorometer (Invitrogen, USA) and electrophoresis on a 0.6% agarose gel. All experiments were performed in accordance with the guidelines of the Animal Ethics Committee and were approved by the Institutional Review Board on Bioethics and Biosafety of BGI.

### MSG library construction and sequencing

Genomic DNA of 144 individuals (142 offspring and 2 parents) were used to construct the library. We prepared the sequencing library using multiplexed shotgun sequencing method proposed by Andolfatto *et al*. [13], with some modifications. Genomic DNA of each sample (1 μg) was digested with 1 μl of FastDigest TaqI restriction enzyme (Thermo Scientific, USA) in FastDigest buffer for 10 minutes at 65°C in a total volume of 30 μl. Barcode adapters were designed and modified according to the standard Illumina adapters designed for paired-end read libraries. Unique barcode adapters (10 μmol) were added to each sample well. The ligation reaction was incubated for 1 hour at 22°C with 2 μl of T4 DNA ligase (Enzymatics, USA), 4 μl of 10× ligase buffer, 30 μl of digested products and 2.5 μl H_2_O. The T4 ligase was heat deactivated at 65°C for 20 minutes. Twenty-four ligation products of different samples were pooled in a single tube, and then 2 μl of chloroform was added to inactivate the restriction enzyme. DNA fragments were purified by excising a DNA fraction of 400–600 bp on a 2% agarose gel and then extracted by a QIA quick Gel Extraction Kit (Qiagen, Germany). All the products were amplified with 10 cycles of PCR in 50-μl reactions with 1 μl of common primer (10 μM), 1 μl of index primer and 25 μl of Phusion Master Mix (Finnzymes, Thermo Scientific, USA). The PCR profile was as follows: initial 30 s at 98°C, then 10 cycles, each with 30 s DNA denaturation at 98°C, 30 s at the annealing temperature 65°C and 30 s extension at 72°C, and a final extension of 5 min at 72°C. The PCR amplicons of 450–750 bases were size extracted using gel electrophoresis (0.5 × TAE, 2% agarose), subsequently purified using a QIAquick PCR Purification Kit (Qiagen), and quantified on Agilent 2100 Bioanalyzer (Agilent, USA). Sequencing was performed on the Illumina Hiseq 2000 platform (Illumina, USA) in 90-bp pair-end reads following standard protocols. Four lanes in the Illumina Hiseq 2000 were used.

### Raw data filter and barcode reads split

Sequence reads from the Illumina runs were filtered as follows: reads with ambiguous base calls (N) more than ten percent, excessive low-quality positions (>40 positions with quality <2) were removed. The remaining trimmed, high-quality reads formed the basis for all subsequent analysis. Sequence reads from the same library were then sorted into individuals by barcode. Meanwhile reads with a barcode that did not match one of the expected barcodes (i.e. a sequencing error in the barcode) were discarded.

### Genotyping

Retained reads were sorted into loci and genotyped using Stacks software (Version 0.9996) to analyze these maps cross data [15]. The Stacks software package is freely downloadable at http://creskolab.uoregon.edu/stacks/. We used Stacks to identify loci in a set of individuals, de novo, and then genotype each locus. Stacks incorporates the likelihood-based SNP calling algorithm (maximum likelihood statistical model), which can evaluate each nucleotide position in every tag of all individuals, and then to identify sequence polymorphisms and distinguish them from sequencing errors [15,16]. Some tag genotypes contained a single SNP, but others represented alleles that differed by multiple SNPs that were scored from these haplotypes.

Markers segregated in four different patterns. Type nn × np (1:1) was homozygous in the male and heterozygous in the female; lm × ll (segregating 1:1) was heterozygous in the male parent and homozygous in the female parent; hk × hk (1:2:1) was heterozygous in both parents with two shared alleles; and ef × eg (1:1:1:1) was heterozygous in both parents with two sex-specific alleles and one shared allele.

### Selection and acquisition of high-quality SNP markers

As mentioned above, not every tag genotype contained a single SNP. In fact, some contained multiple SNPs. We selected SNPs for our further genetic map construction according to haplotypes identified by the Stacks genotypes program output using our custom scripts. The SNP markers were firstly filtered by excluded loci that genotype call rate less than 95%, and then a Chi-square goodness-of-fit test was used to assess the Mendelian segregation patterns. Those SNP markers showing significant segregation distortion (*χ*^2^ test, P < 0.001) were discarded.

### Genetic linkage map construction

Linkage groups (LGs) were established by JoinMap v4.0 [17]. The linkage map was built using the regression mapping algorithm, a recombination frequency smaller than 0.4, and an independence LOD threshold of 8.0. Recombination frequencies were converted to centiMorgans (cM) using Kosambi’s method for map-distance calculation, and the LGs maps were drawn and aligned using MapChart v2.2 [18].

### Sequence comparison

Consensus sequences of the mapped MSG tag (84 bases in length) were aligned with the genomic sequences of five other fish species. The zebrafish *Danio rerio* (Zv9), three-spined stickleback *Gasterosteus aculeatus* (ver. 1.0), medaka *Oryzias latipes* (ver. 1.0), tilapia *Oreochromis niloticus* (ver. 1.0) and fugu *Takifugu rubripes* (ver. 5.0) genome sequences were downloaded, and blastn (BLAST + ver. 2.2.21) [19] searches with an e-value cutoff of 10^-10^ were conducted. In cases where the search of a query sequence hit two or more loci, a hit with the smallest e-value was considered significant. Significant hits on the chromosomes were used, including unoriented scaffolds assigned to chromosomes in the fugu genome. The Oxford grids [20] were constructed to study synteny and to compare positions of the homologous loci using Grid Map ver. 3.0a (http://cbr.jic.ac.uk/dicks/software/Grid_Map/).

## Results

### Sequencing and genotyping

The Hiseq 2000 sequencing yielded 594,142,945 90-base reads. We generated more than 93 Gb of clean sequence data after excluded low quality raw reads. The sequences have been submitted to DDBJ under the accession number PRJDB1493. The average count of MSG tags per individual was 7,694,515. The mean coverage depth of MSG tags is 17.4 ×. MSG tags were aligned and clustered into 423,943 stacks, and 58,708 candidate MSG loci were inferred. For the analysis of the F1 mapping population, 25,892 SNPs were informative and were scored for sufficient numbers of progeny. Among them, 18,256 SNPs were retained after discarding those with a deviation from a Mendelian segregation pattern, and they were passed forward into the linkage map construction. The sequence dataset for this study was shown in the Additional file 1.

### Genetic linkage map

**Figure 1:**
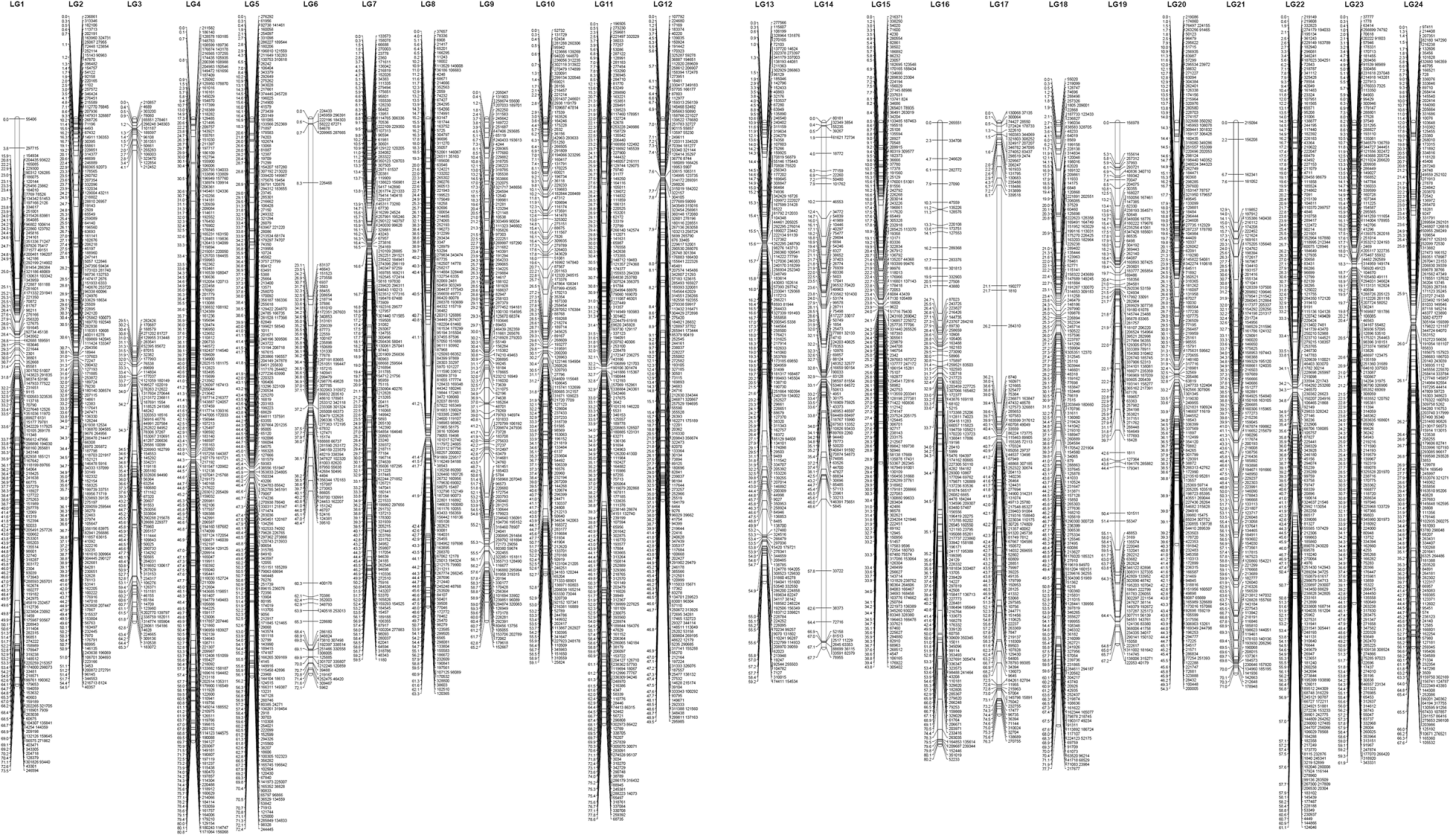
**The sex-averaged linkage map of orange-spotted grouper.** The lengths of the linkage groups are based on Kosambi cM.

Linkage analysis identified 24 linkage groups (LG1-LG24), which is consistent with the haploid chromosome number of the orange-spotted grouper [21]. The sex-averaged map (Figure 1) contained a total of 4,608 SNPs, which spanned 1581.7 cM, with a mean distance between SNPs of 0.34 cM. The number of SNPs mapped on a linkage group of the sex-averaged map ranged from 115 (LG14) to 250 (LG5); the lengths of the linkage groups ranged from 43.8 (LG15) to 80.8 cM (LG4) (see details in Table 1). However, distribution of markers was uneven, likely reflecting differences in recombination frequency along the length of the grouper chromosomes. Clustered marker regions were observed in every linkage group of the sex-averaged map, especially in positions close to the centromeres and, less frequently, at the telomeres. To characterize marker clustering, we tabulated the number of markers sharing an identical genetic map location with other markers, as well as the number of unique location. The 4,608 SNPs were located in 2849 unique locations on the linkage map, with an average inter-location space of 0.56 cM (Table 1).

**Table 1:** Summary of the genetic linkage maps of orange-spotted grouper

**Linkage group**	**Number of SNPs**			**Number of unique loci**			**Linkage group size (cM)**			**Recombination rates (famale:male)**	**cM/SNPs**			**cM/loci**		
	**Sex-averaged**	**Female**	**Male**	**Sex-averaged**	**Female**	**Male**	**Sex-averaged**	**Female**	**Male**		**Sex-averaged**	**Female**	**Male**	**Sex-averaged**	**Female**	**Male**
LG1	198	92	121	122	72	87	73.5	56.3	61.7	0.91	0.37	0.61	0.51	0.60	0.78	0.71
LG2	205	116	121	133	88	86	54.5	57.5	50.8	1.13	0.27	0.50	0.42	0.41	0.65	0.59
LG3	122	54	104	77	34	75	65.3	57.0	54.9	1.04	0.54	1.06	0.53	0.85	1.68	0.73
LG4	240	129	143	159	91	96	80.8	72.4	54.3	1.33	0.34	0.56	0.38	0.51	0.80	0.57
LG5	250	126	123	166	77	84	72.4	52.3	58.0	0.90	0.29	0.42	0.47	0.44	0.68	0.69
LG6	123	99	119	68	69	87	72.2	52.8	57.2	0.92	0.59	0.53	0.48	1.06	0.77	0.66
LG7	186	102	106	125	85	81	59.5	62.1	56.7	1.10	0.32	0.61	0.53	0.48	0.73	0.70
LG8	223	129	156	99	101	89	63.3	58.6	53.1	1.10	0.28	0.45	0.34	0.64	0.58	0.60
LG9	166	142	134	115	91	98	65.2	62.2	60.2	1.03	0.39	0.44	0.45	0.57	0.68	0.61
LG10	183	93	136	130	74	91	59.1	64.1	49.0	1.31	0.32	0.69	0.36	0.45	0.87	0.54
LG11	234	94	135	168	75	90	78.6	53.6	59.4	0.90	0.34	0.57	0.44	0.47	0.71	0.66
LG12	247	76	135	109	54	80	49.5	58.4	46.2	1.26	0.20	0.77	0.34	0.45	1.08	0.58
LG13	208	131	152	112	88	86	59.2	55.5	57.8	0.96	0.28	0.42	0.38	0.53	0.63	0.67
LG14	115	109	107	75	90	74	67.7	55.6	49.8	1.12	0.59	0.51	0.47	0.90	0.62	0.67
LG15	203	110	126	131	97	78	43.8	55.1	52.0	1.06	0.22	0.50	0.41	0.33	0.57	0.67
LG16	165	111	105	94	90	81	80.2	59.1	65.3	0.91	0.49	0.53	0.62	0.85	0.66	0.81
LG17	144	101	137	88	90	84	76.3	58.0	61.4	0.94	0.53	0.57	0.45	0.87	0.64	0.73
LG18	193	125	107	144	117	75	77.7	65.4	53.5	1.22	0.40	0.52	0.50	0.54	0.56	0.71
LG19	156	57	116	72	37	84	67.2	45.3	63.4	0.71	0.43	0.79	0.55	0.93	1.22	0.75
LG20	199	75	80	134	61	60	54.3	51.7	48.5	1.07	0.27	0.69	0.61	0.41	0.85	0.81
LG21	143	131	146	109	88	108	71.0	55.5	53.0	1.05	0.50	0.42	0.36	0.65	0.63	0.49
LG22	249	130	91	150	85	67	61.1	50.5	56.6	0.89	0.25	0.39	0.62	0.41	0.59	0.84
LG23	221	75	115	156	60	79	61.9	53.5	53.9	0.99	0.28	0.71	0.47	0.40	0.89	0.68
LG24	235	109	124	113	88	85	67.6	58.4	59.1	0.99	0.29	0.54	0.48	0.60	0.66	0.70
Total	4608	2516	2939	2849	1902	2005	1581.7	1370.9	1335.5	1.03	0.34	0.54	0.45	0.56	0.72	0.67

Sex specific maps (Additional file 2: Figure S1 and Additional file 3: Figure S2) were also constructed. There were 2,516 SNPs on the female map, and the number of unique locus was 1,902. The length of the female map was 1370.9 cM, with the average inter-SNP distance and inter-location space at 0.54 and 0.72 cM respectively. The male map contained 2,939 SNPs, and there were 2,005 unique locations. The length of the male map was 1335.5 cM, and the mean length between SNPs and locations was 0.45 and 0.67 cM respectively. The female map comprised linkage groups ranging in length from 45.3 to 72.4 cM while the male map contained linkage groups with a length ranging from 46.2 to 65.3 cM (Table 1).

The differences in recombination rates between the sexes are presented in Table 1. The overall recombination rate between female and male is 1.03, and the rates among different linkage groups range from 0.71 to 1.33.

### Syntenies between different fish species

BLAST searches of the 5,194 mapped MSG tag consensus sequences from the orange-spotted grouper against the genome sequences of tilapia, stickleback, fugu, medaka and zebrafish indicated variation in the syntenic relationship between orange-spotted grouper and the respective species. Homology was most frequently inferred to the tilapia genome, with 225 tags being mapped to it. In contrast, the other four species yielded limited numbers of similarity hits. Only 177, 83, 72 and 15 tags mapped to the stickleback, fugu, medaka and zebrafish genome sequences, respectively. Owing to the tilapia genome sequence have not been integrated with linkage map, the syntenic relationship of linkage group between orange-spotted grouper and tilapia could not be evaluated. Detailed syntenic pairs between orange-spotted grouper linkage groups and four model fish species chromosomes were show on Additional file 4: Figure S3.

## Discussion

### MSG and linkage mapping

In this study, 25,892 SNPs were informative and were scored for sufficient numbers of progeny, and then a Chi-square goodness-of-fit test (*χ*2 test, P < 0.001) was used to assess these SNPs whether deviate from the Mendelian segregation patterns. 18,256 SNPs were retained after discarding those with a deviation from a Mendelian segregation pattern, suggesting non-monogenetic inheritance of those discarded SNP markers. Technical artifacts may be responsible for the distorted markers, but biological processes known as transmission ratio distortion (TRD) also cause a deviation from Mendelian segregation [22]. Both regression and ML mapping algorithm in JoinMap4.0 can be used to arrange markers. More SNPs can be mapped base on the ML mapping algorithm (data not shown). However, when we evaluated the results by aligning the SNPs that arranged on the maps to the genome of *E. coioides* (genome data not published), we found the order of SNPs based on the regression mapping algorithm was more reliable. So we choose the regression mapping algorithm. Finally, a high-density linkage map which contained a total of 4,608 SNPs and spanned 1581.7 cM, with a mean distance between SNPs of 0.34 cm was obtained. Such highly dense linkage maps contain rich information on the genomic structure of an organism and could be useful for studies involving comparative genomics and QTL mapping.

To date, microsatellite marker has been a popular option for linkage analyses in organisms without genomic information, especially for aquatic animals [23,24]. Microsatellite markers are sequence-based, but they are costly and time-consuming if hundreds or thousands of markers are involved [22]. In contrast to microsatellite markers, SNPs generated by MSG are abundant and highly suitable for cost effective high-throughput genotyping. These SNPs are sequence-based, allowing the practice of comparative genomics [22,25], which aids in exploring candidate genes for traits of interest [26] and even assembling *denovo* genomic sequences [27]. Moreover, allelic information on a large number of SNPs is readily available without prior curation and labourious experiments. The present study further demonstrates the utility of MSG in the genomic study of a non-model organism, yielding a wealth of genomic information without prior knowledge of the genome of an examined species.

The utility of genetic maps is correlated with the distribution of markers across linkage groups. Clustered markers in areas of minimal recombination, while allowing general linkage group assignment, often cannot be used for definitive fine mapping and positional cloning [5]. In fish, clustering of DNA markers on genetic linkage maps has been observed in medaka [28], rainbow trout [29], channel catfish [5,30], and Atlantic salmon [31]. Although potential explanations for high levels of marker clustering are not completely understood [5,32], large areas of repetitive DNA in teleost genome result from whole genome duplication could be the important reason for this phenomenon.

### Differences in recombination rates between the male and female

It is common to find a difference in the recombination rate between the two sexes in any fish species with female map distances usually larger than those in male map. For instance, the female:male recombination rate was 1.37:1 in Atlantic salmon [4], 1.68:1 in rainbow trout [6], 1.43:1 in Japanese flounder [8] and 1.6:1 in catfish [5,33]. In contrast to these previous reports, the linkage maps presented in this study (Table 1) show a smaller overall recombination rate between female and male (1.03:1). The reason for this remarkable difference in reported recombination rates is likely to be our maps improved marker coverage in telomeric regions. Since male recombination is often elevated in telomeres [34,35], the more comprehensive coverage of these regions in our study has resulted in a more even recombination rate between sexes. Thus, it is quite likely that the male and female map lengths will converge further when marker density is increased.

### Comparative genome analysis

The sequences of 225, 177, 83, 72 and 15 mapped markers had significant hits in the whole genome sequences of tilapia, stickleback, fugu, medaka and zebrafish, respectively, suggesting that tilapia is more closely related to grouper than the other four fishes.

Linkage map with sequence-based markers is a platform for comparative genome studies. It is well known that determination of gene functions is difficult in non-model species; functional genome analysis will have to rely heavily on the establishment of homologies from model species. Mapping more gene sequences on the linkage map of the orange-spotted grouper should enhance comparative mapping, thereby transferring genome information from model species to the orange-spotted grouper.

## Conclusions

We constructed highly dense genetic linkage maps of the orange-spotted grouper using MSG. The number of linkage groups is consistent with the haploid chromosome number of the orange-spotted grouper.

This study presents the high density genetic linkage maps for the groupers. It was produced from genotypes of 142 F1 full-sib progeny and included 4608 SNP markers. The sex-averaged map revealed 24 linkage groups that covered 1581.7 cM, with an average intermarker distance of 0.34 cM. These high density maps allowed for comparison among them and those from model fish species, and will be useful for research on grouper genetics and molecular breeding.

## Competing interests

The authors declare that they have no competing interests.

## Authors’ contributions

QS, XY, YZ and HL initiated and supervised the study. LJ, GC, HZ and YZ prepared the F1 full-sib progeny of orange-spotted grouper. JC, JL, JB, QM, QX, QC and YC performed the experiments. LS, XY and SL conducted data analysis. XY, LS and QS prepared the manuscript. All authors read and approved the final manuscript.

## Supplementary Material

Additional file 1 List of MSG stack sequences, including consensus sequences of MSG loci. Click here for file

Additional file 2: Figure S1 The female linkage map of orange-spotted grouper. The lengths of the linkage groups are based on Kosambi cM. Click here for file

Additional file 3: Figure S2 The male linkage map of orange-spotted grouper. The lengths of the linkage groups are based on Kosambi cM. Click here for file

Additional file 4: Figure S3 Oxford grids between genome of orange-spotted grouper and four model fishes. Each number in a cell denotes the number of homologous pair of loci in each genome. The homologous loci were inferred from sequence similarity searches of mapped MSG-tags against the genome sequences of model fishes. Cells with more than one pair are highlighted in yellow. Click here for file
