# Supplementary material for: Construction of high-density genetic linkage maps for orange-spotted grouper Epinephelus coioides using multiplexed shotgun genotyping
Source: BMC Genet. 2013 Dec 1;14:113. doi: 10.1186/1471-2156-14-113 (PMC3890575; doi:10.1186/1471-2156-14-113)
Supplement: Additional file 3: Figure S2 — The male linkage map of orange-spotted grouper. The lengths of the linkage groups are based on Kosambi cM. [file 1471-2156-14-113-S3.pdf]

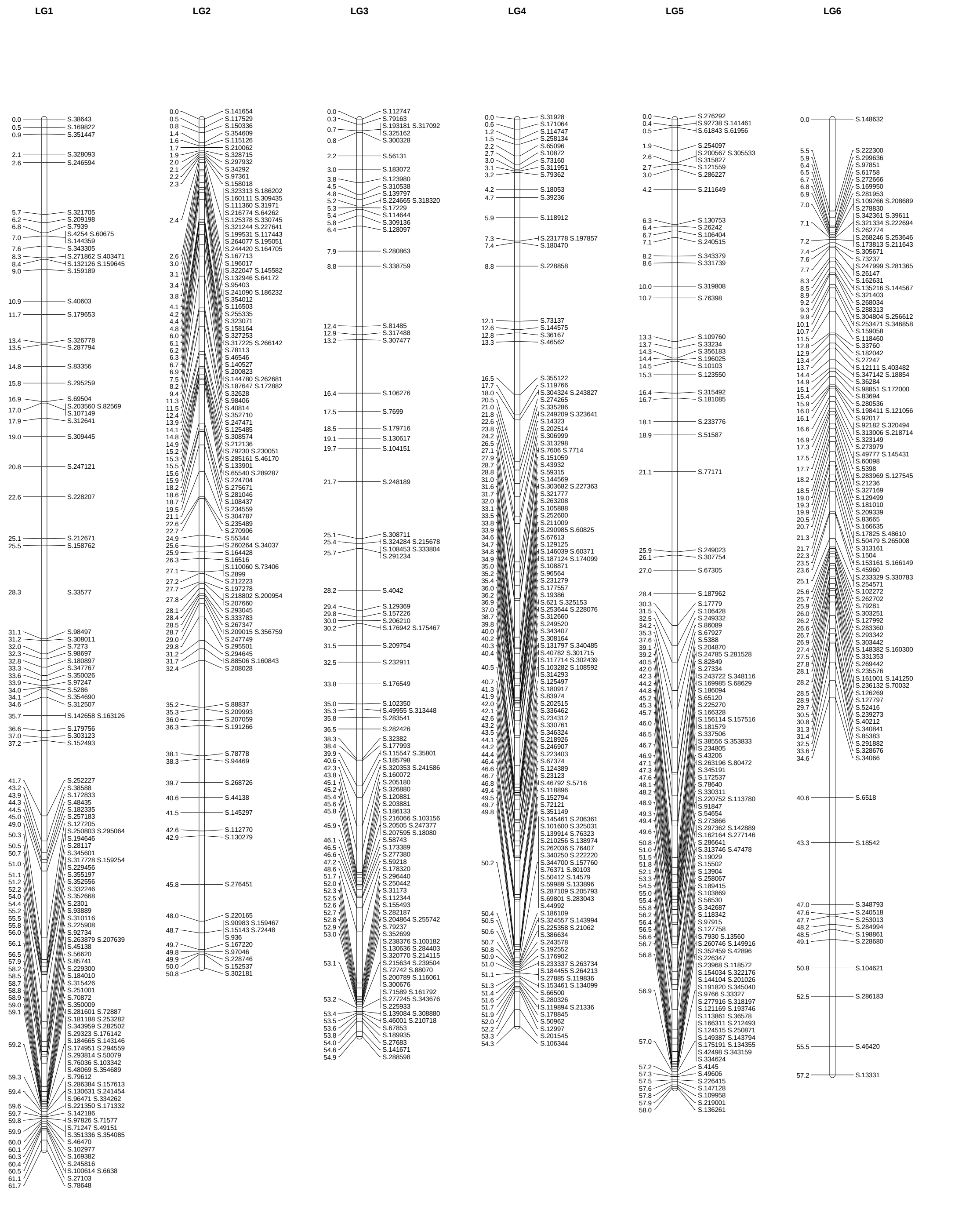

Figure S2. The male linkage map of orange-spotted grouper. The lengths of the linkage groups are based on Kosambi cM.

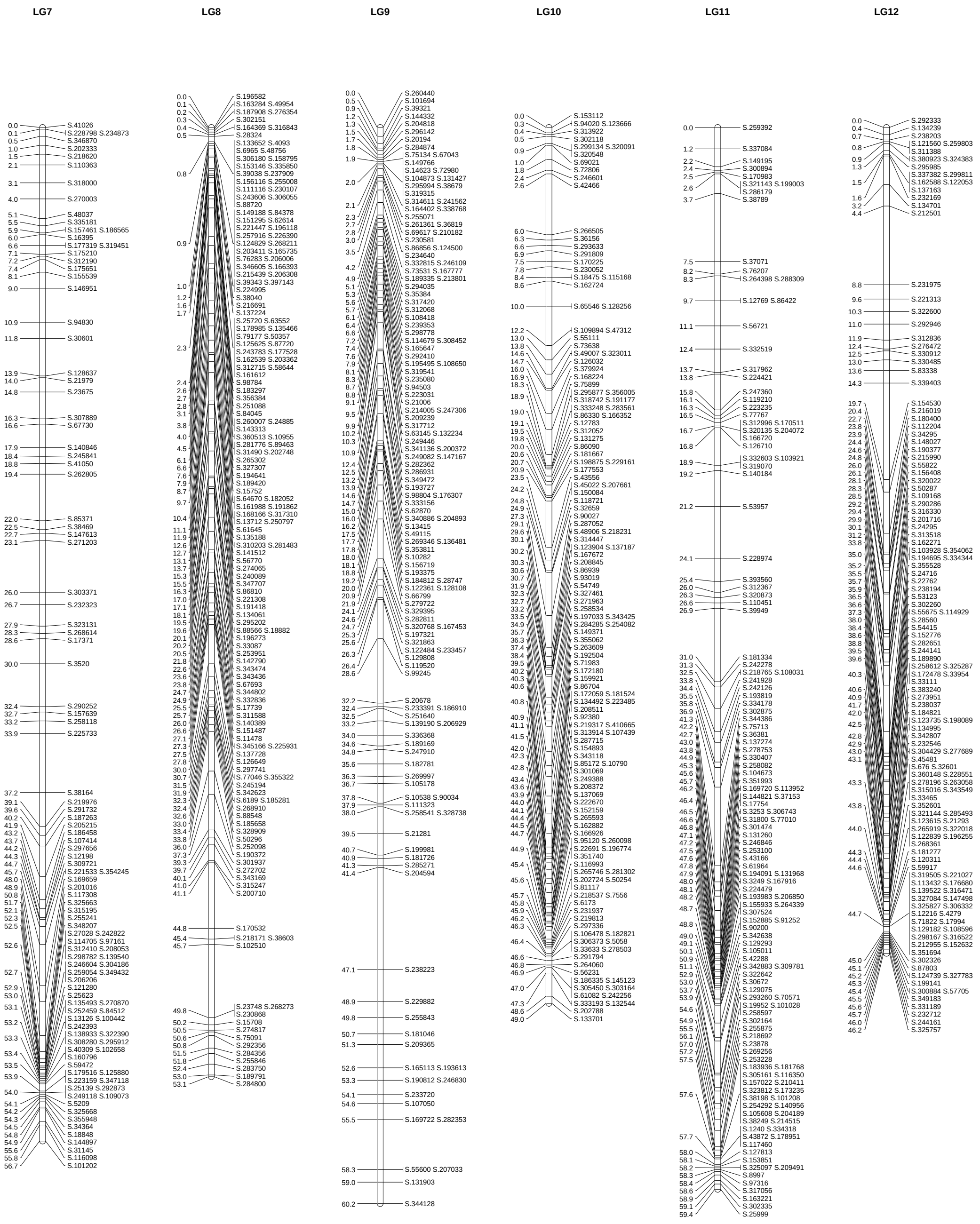

Figure S2 (continued)

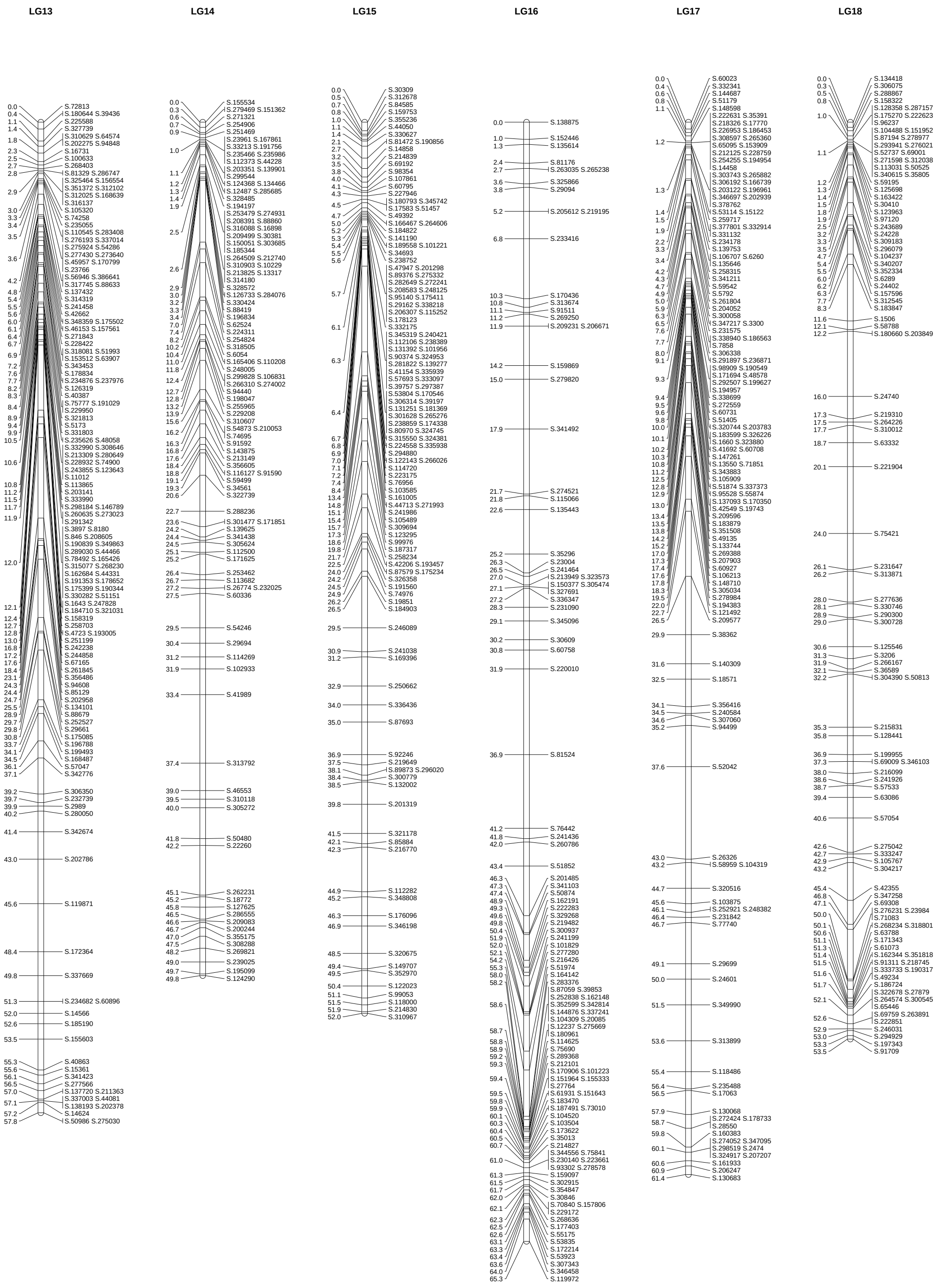

Figure S2 (continued)

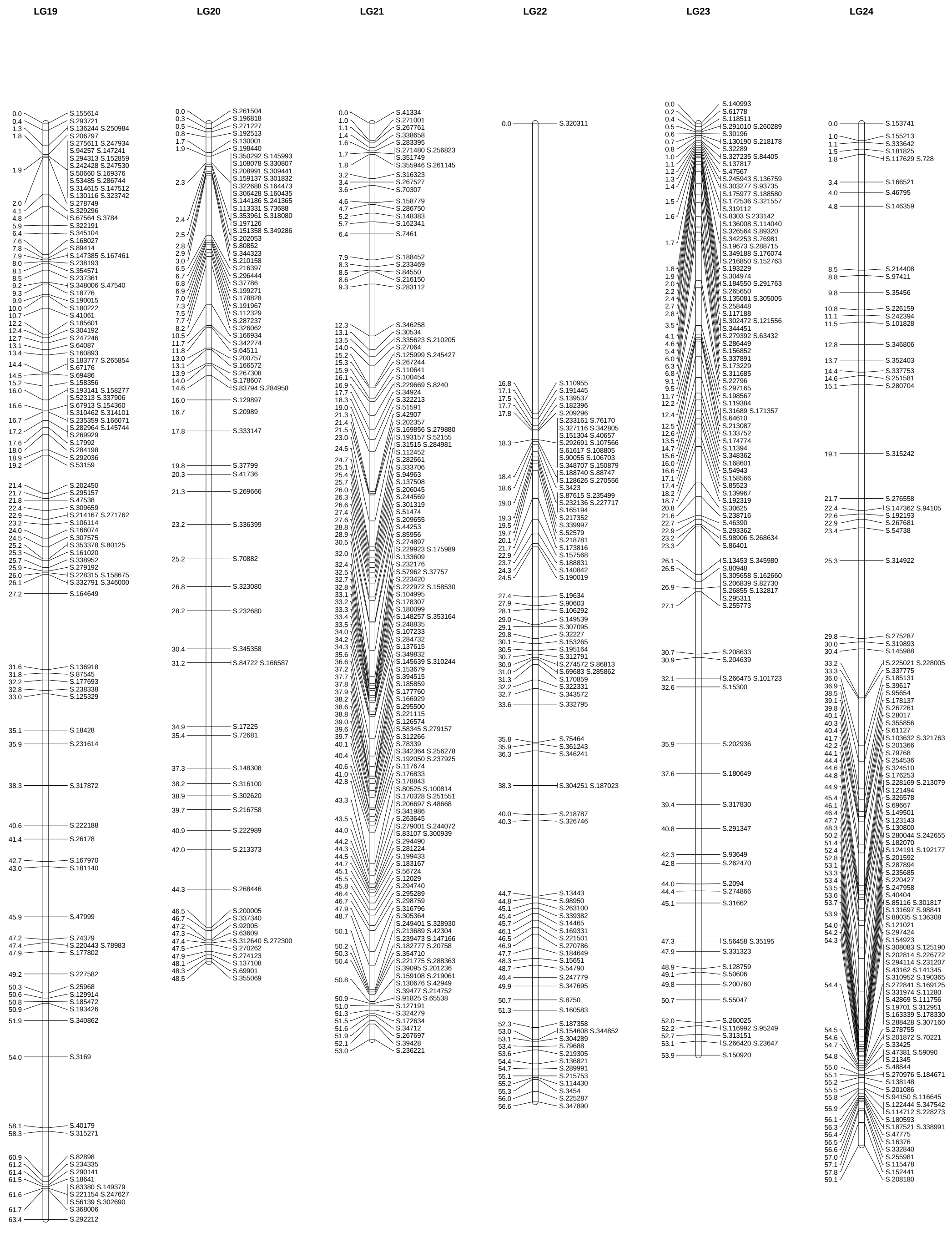

Figure S2 (continued)
