# Supplementary material for: Construction of high-density genetic linkage maps for orange-spotted grouper Epinephelus coioides using multiplexed shotgun genotyping
Source: BMC Genet. 2013 Dec 1;14:113. doi: 10.1186/1471-2156-14-113 (PMC3890575; doi:10.1186/1471-2156-14-113)
Supplement: Additional file 4: Figure S3 — Oxford grids between genome of orange-spotted grouper and four model fishes. Each number in a cell denotes the number of homologous pair of loci in each genome. The homologous loci were inferred from sequence similarity searches of mapped MSG-tags against the genome sequences of model fishes. Cells with more than one pair are highlighted in yellow. [file 1471-2156-14-113-S4.zip › Additional files 4/Figure S3-3.pptx]

## Slide 1
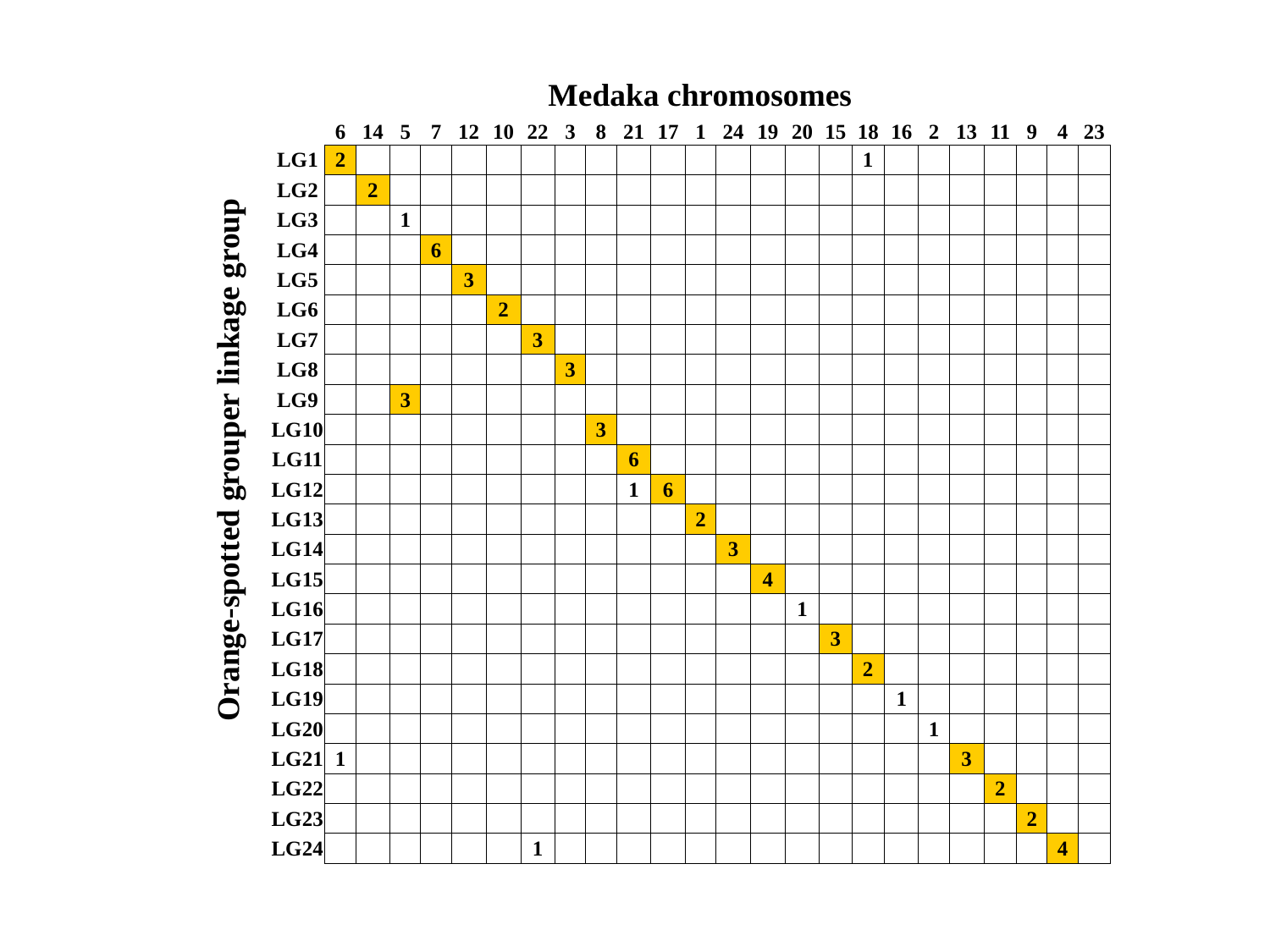

Medaka chromosomes
| | 6 | 14 | 5 | 7 | 12 | 10 | 22 | 3 | 8 | 21 | 17 | 1 | 24 | 19 | 20 | 15 | 18 | 16 | 2 | 13 | 11 | 9 | 4 | 23 |
| --- | --- | --- | --- | --- | --- | --- | --- | --- | --- | --- | --- | --- | --- | --- | --- | --- | --- | --- | --- | --- | --- | --- | --- | --- |
| LG1 | 2 | | | | | | | | | | | | | | | | 1 | | | | | | | |
| LG2 | | 2 | | | | | | | | | | | | | | | | | | | | | | |
| LG3 | | | 1 | | | | | | | | | | | | | | | | | | | | | |
| LG4 | | | | 6 | | | | | | | | | | | | | | | | | | | | |
| LG5 | | | | | 3 | | | | | | | | | | | | | | | | | | | |
| LG6 | | | | | | 2 | | | | | | | | | | | | | | | | | | |
| LG7 | | | | | | | 3 | | | | | | | | | | | | | | | | | |
| LG8 | | | | | | | | 3 | | | | | | | | | | | | | | | | |
| LG9 | | | 3 | | | | | | | | | | | | | | | | | | | | | |
| LG10 | | | | | | | | | 3 | | | | | | | | | | | | | | | |
| LG11 | | | | | | | | | | 6 | | | | | | | | | | | | | | |
| LG12 | | | | | | | | | | 1 | 6 | | | | | | | | | | | | | |
| LG13 | | | | | | | | | | | | 2 | | | | | | | | | | | | |
| LG14 | | | | | | | | | | | | | 3 | | | | | | | | | | | |
| LG15 | | | | | | | | | | | | | | 4 | | | | | | | | | | |
| LG16 | | | | | | | | | | | | | | | 1 | | | | | | | | | |
| LG17 | | | | | | | | | | | | | | | | 3 | | | | | | | | |
| LG18 | | | | | | | | | | | | | | | | | 2 | | | | | | | |
| LG19 | | | | | | | | | | | | | | | | | | 1 | | | | | | |
| LG20 | | | | | | | | | | | | | | | | | | | 1 | | | | | |
| LG21 | 1 | | | | | | | | | | | | | | | | | | | 3 | | | | |
| LG22 | | | | | | | | | | | | | | | | | | | | | 2 | | | |
| LG23 | | | | | | | | | | | | | | | | | | | | | | 2 | | |
| LG24 | | | | | | | 1 | | | | | | | | | | | | | | | | 4 | |
Orange-spotted grouper linkage group
